# Multidisciplinary population monitoring when demographic data are sparse: a case study of remote trout populations

**DOI:** 10.1002/ece3.871

**Published:** 2013-11-08

**Authors:** Dylan J Fraser, Anna M Calvert, Louis Bernatchez, Andrew Coon

**Affiliations:** 1 Department of Biology, Concordia University7141 Sherbrooke St. West, Montreal, QC, H4B 1R6, Canada; 2 Département de Biologie, Institut de Biologie Intégrative et des Systèmes (IBIS), Université LavalPavillon Charles-Eugène-Marchand 1030, Avenue de la Médecine Local 1145, Québec, QC, G1V 0A6, Canada; 3 Tourism Office, Cree Nation of MistissiniMistissini, QC, G0W 1C0, Canada

**Keywords:** effective population size, genetic monitoring, life history, Mistassini Lake, salmonid, traditional ecological knowledge.

## Abstract

The potential of genetic, genomic, and phenotypic metrics for monitoring population trends may be especially high in isolated regions, where traditional demographic monitoring is logistically difficult and only sporadic sampling is possible. This potential, however, is relatively underexplored empirically. Over eleven years, we assessed several such metrics along with traditional ecological knowledge and catch data in a socioeconomically important trout species occupying a large, remote lake. The data revealed largely stable characteristics in two populations over 2–3 generations, but possible contemporary changes in a third population. These potential shifts were suggested by reduced catch rates, reduced body size, and changes in selection implied at one gene-associated single nucleotide polymorphism. A demographic decline in this population, however, was ambiguously supported, based on the apparent lack of temporal change in effective population size, and corresponding traditional knowledge suggesting little change in catch. We illustrate how the pluralistic approach employed has practicality for setting future monitoring efforts of these populations, by guiding monitoring priorities according to the relative merits of different metrics and availability of resources. Our study also considers some advantages and disadvantages to adopting a pluralistic approach to population monitoring where demographic data are not easily obtained.

## Introduction

For practical reasons, traditional demographic monitoring of populations is increasingly complemented with genetic and evolutionary approaches (Hansen et al. 2006, 2012; Schwartz et al. 2007; Hendry et al. 2011). Particularly when populations are small, or species are elusive, difficult to capture, or leave their wastes behind, genetic approaches can be less resource intensive for estimating population abundance and growth (Muracco et al. 2009; Antao et al. 2010; De Barba et al. 2010; Tallmon et al. 2012). Even when populations are large, metrics of genetic, genomic, or phenotypic change can signal that demographic decline has occurred or might occur (Hendry et al. 2011; Jakobsdottir et al. 2011; Hansen et al. 2012; Côté et al. 2013), although their explicit links with demographic change are in general more difficult to confirm (Cuveliers et al. 2011; Osborne et al. 2012; Tallmon et al. 2012).

Populations of species found in many of the world's remaining isolated regions are experiencing increasing exploitation or other human disturbances, and they pose a challenge to population monitoring. On the one hand, traditional demographic monitoring is difficult if not impossible in such regions for economic and logistic reasons; sampling can be conducted only intermittently or seasonally (Ferguson and Messier 1997; Berkes 1999; Fraser et al. 2006). On the other hand, while sampling of genetic, genomic, and phenotypic metrics is more feasible (e.g., Fraser and Bernatchez 2005; Gomez-Uchida et al. 2012), such regions are also more likely to harbor the last remaining population strongholds of the focal species. Thus, changes in these metrics can be more difficult to interpret with respect to moderate or large population demography (Tallmon et al. 2012). Overall, while empirical assessments of such metrics for monitoring population trends are rapidly accumulating (e.g., De Barba et al. 2010; Hansen et al. 2012; Osborne et al. 2012), we still have much to learn, and few examples come from populations from isolated regions.

The present multidisciplinary study assesses several metrics of population health which are amenable to monitoring in isolated regions, using socioeconomically important freshwater fish populations. It then considers the advantages and disadvantages of such a pluralistic approach for making management recommendations, given the incomplete nature and/or potential bias of different metrics that might arise where sampling can only be carried out sporadically. The rationale behind adopting each of our study's monitoring metrics is described below. Our study deals with a common situation where exploited freshwater fishes include multiple populations that are genetically, morphologically, and ecologically differentiated (Taylor 1999). Monitoring and maintaining this population diversity has important practical implications as it may be linked to long-term species persistence, increased yield, and reduced annual variability in productivity (Schindler et al. 2010).

First Nations peoples of northern Canada have long depended on the harvesting of freshwater fish populations for their subsistence and well-being (Berkes 1999). The Cree of the Mistassini Lake region, Quebec's largest postglacial lake (2150 km^2^), is no exception. Their demand for these fish has increased in the past decade with a 31.4% increase in the local human population (2597–3427 people from 2001 to 2011; Statistics Canada 2012). Recent decades have also seen a steady increase in the development of regional mining infrastructure, tourism infrastructure associated with seasonal fishing camps, and public access resulting from the expansion of the only road in the region. Due to the large size of captured individuals, Mistassini Lake's brook trout (*Salvelinus fontinalis,* Mitchill) are among Quebec's and eastern North America's most sought after fish by subsistence fishers and sport fishers.

Mistassini Lake's discharge, the Rupert River (hereafter abbreviated RUP), and its northeast tributaries, the Cheno (CHE) and Pepeshquasati (PEP) Rivers, are historically known as the main spawning grounds for adult brook trout and as nurseries for juveniles. Each river harbors a genetically distinct population comprised of individuals that migrate as juveniles to lake feeding areas for one to four years before returning, predominantly to the same river, to spawn and complete the life cycle (Fraser et al. 2004; Fraser and Bernatchez 2005). Each population also contributes differentially to the annual harvest throughout the lake (Fraser and Bernatchez 2005). Yet between 1970 and 2000, Cree fishers anecdotally reported an increase in the average time required to catch a trout and a decrease in the number of trout captured (Fraser et al. 2006). Whether or not such trends persist to the present day and whether they are representative of all three populations are of interest given increasing anthropogenic influences in the region in the past decade, and given that some data from 2000 to 2002 have suggested Mistassini's populations were not highly abundant (Fraser et al. 2004).

Our study compared data on contemporary Mistassini brook trout populations (2011) with archival data collected in 2000–2002. We specifically assessed changes in the following:

1. *Catch-per-unit effort (CPUE)*. While changes in standardized catch rates of fish are, at best, an indirect proxy of abundance, reductions in CPUE may reflect a decline in adult abundance (Harley et al. 2001).
2. *Life-history characteristics*. Specific life-history traits of interest were age and size (length) composition of breeding adults. Fluctuations in these traits can be environmentally driven, but their reductions are often attributed to overfishing over short timescales. Subsequently, these changes may negatively influence population growth and persistence (Jorgensen et al. 2007; Hutchings and Fraser 2008).
3. *Genetic and genomic diversity*. Of particular interest was the level of genetic diversity and extent of genetic change exhibited between time periods, as these can be indicators of changes in population size (Leberg 2002; Schwartz et al. 2007; Côté et al. 2013). Allelic frequency changes at loci under selection may also be linked to human activities, such as overharvesting (Nielsen et al. 2009).
4. *Estimation of the number of breeding trout in each population*. We wanted to determine whether numbers of breeding trout within each population were stable, increasing, or decreasing between 2000–2002 and 2011. This has direct implications for setting appropriate harvesting levels in Mistassini Lake (Fraser et al. 2006), yet directly estimating population size was extremely difficult due to the lake's remote location and large size. We therefore focused on estimating effective population sizes (*N*_*e*_) as changes in *N*_*e*_ can provide an indication of shifts in the number of individuals contributing offspring to the next generation.
5. *Cree traditional ecological knowledge**.*** Of chief interest was the current status of populations relative to a decade ago (Fraser et al. 2006) as evaluated by local Cree fishers, with specific focus on the spatial distribution of trout, trout catches, and local conservation concerns.

## Materials and Methods

### Fish sampling, catch-per-unit-effort and life-history analyses

**Figure 1:**
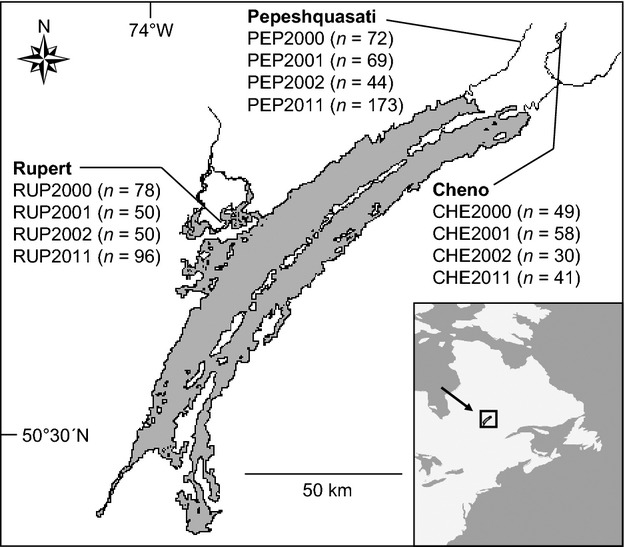
Sampling locations of spawning populations of brook trout in Mistassini Lake, Quebec, as well as the number of trout sampled per population per year of the study for microsatellite analyses. Modified and updated from Fraser et al. (2004).

A total of 810 prespawning brook trout were captured via angling from multiple locations and times within CHE, PEP, and RUP in the fall (September 15 to October 15) of 2000, 2001, 2002, and 2011 (Fig. 1). Within populations, the same sampling regime was applied each year, using (i) the same time of year (within 1–1.5 weeks); (ii) approximately the same number of sampling days; (iii) the same and large number of spatial locations; (iv) the same angling techniques; (v) the same time of day (9.00 h–17.00 h); (vi) the same number of anglers; and (vii) in most cases, the same anglers.

Sampling consisted of (i) determining the sex, age, and total length (mm) of each trout; (ii) collecting a small piece of adipose fin tissue for DNA analyses; and (iii) calculating the number of trout captured per eight-hour day of fishing per angler (CPUE). Male and female trout were easily discerned from one another based on external morphological characteristics. Age was assessed from standard scale analysis for all trout sampled in 2011 (*n* = 310) and for a subset of trout sampled in 2000–2002 (CHE *n* = 50; PEP *n =* 49; RUP *n* = 43). Age was defined as the number of completed winter seasons (e.g., 2+, 3+, 4+, 5+, 6+, or 7+) and was assessed independently by two different people; 91% the estimates were congruent, while the remaining 9% of estimates differed by only one year and were reassessed a second time. Most trout were released unharmed following sampling; the remainder were killed for consumption by aboriginal fishers.

We used two-factor generalized linear models (GLMs) to compare CPUE, length, age, and length-at-age of prespawning trout across time periods between and within populations (i.e., time period and population were fixed effects). Data for these metrics were not normally distributed; GLMs were therefore fitted with different error distributions. Continuous length data and length-at-age data were skewed, and so a gamma distribution was used. Discrete age data and CPUE data were modeled with a Poisson distribution. The Akaike information criterion (AIC; Akaike 1973) was used to select among models (where the lowest AIC value represents the most parsimonious model; Burnham and Anderson 2002), although inference regarding the significance of particular parameters was further informed by nonzero effect sizes.

### Within-population genetic diversity

Total genomic DNA from adipose fin tissue samples taken from each trout was extracted following Fraser et al. (2004). All trout were genotyped at 14 polymorphic microsatellite loci and a subset of trout at 237 SNPs, of which 167 were polymorphic.

#### Microsatellites

For archival samples (years 2000–2002; *n =* 500), genotypes for seven of these loci originated from Fraser et al. (2004): *Sfo18* (Angers et al. 1995), *SfoB52, SfoC86, SfoC129, SfoD75, SfoD91, and SfoD100* (T.L. King, US Geological Survey, unpublished). Remaining loci used were *Sco218, Sco220* (DeHaan and Ardren 2005), *SalE38* (McGowan et al. 2004), *Ssa408* (Cairney et al. 2000), and *SfoC28, SfoC88, SfoC113* (T.L. King, US Geological Survey, unpublished). Polymerase chain reaction (PCR) profiles followed specific loci protocols in Fraser et al. (2004) and Belmar-Lucero et al. (2012). PCR products were separated electrophoretically using a Life Technologies™ 3500 automated sequencer (Life Technologies, Carlsbad, CA), with allele sizes scored based on a fluorescently labeled size standard. This sequencer was different than the one used in Fraser et al. (2004), so allele sizes at seven loci were standardized between time periods by rerunning 10 archival samples from each population. We investigated potential deviations from Hardy–Weinberg equilibrium (HWE) at each locus as well as linkage disequilibrium (between loci pairs) using GENEPOP 3.1 (Raymond and Rousset 1995).

#### SNPs

A subset of individuals from each population was also screened at SNPs developed for brook trout to conduct genome scans below. These SNPs, all located in coding gene regions, have been positioned on a genetic map, tested for association with QTL at many physiological traits (including growth traits), and annotated when feasible (Sauvage et al. 2012a,b). Of 237 SNPs screened, 167 were polymorphic and amplified in over 85% of individuals and showed no deviations from HWE equilibrium across samples due to technical artefacts (6 loci); monomorphic SNPs were excluded from all analyses. Details of SNP development, validation, and sequencing at the Genome Quebec Innovation Center (McGill University, Montreal, QC, Canada) are found in Sauvage et al. (2012a,b). The number of trout screened for SNPs in this study totalled 269: 119 from the archival period, based on year 2000 samples (CHE *n* = 37, PEP *n* = 41, RUP *n* = 41), and 150 from year 2011 samples (CHE *n* = 37, PEP *n* = 57, RUP *n* = 56).

### Temporal analyses of genetic diversity and differentiation

To assess the degree of temporal stability in population structure, we firstly compared allelic diversity and observed heterozygosities between time periods within populations, using GLMs fitted with a Gaussian error distribution (data for both were normally distributed), and based on the model selection procedure described above. We then compared Weir and Cockerham's (1984) *F*_ST_ analogue, *θ*_ST_, between and within populations for all sampling years (using GENETIX 4.05; Belkhir et al. 2004). Finally, an analysis of molecular variance (AMOVA) was performed (using Arlequin 3.0; Excoffier et al. 2005) to assess components of genetic diversity attributable to (i) variance among populations (spatial component); (ii) variance among time periods within populations (temporal component); and (iii) variance among individuals within temporal samples. Archival years 2000–2002 were pooled, as our main interest was potential change between time periods, and because of their temporal stability in Fraser et al. (2004). AMOVAs were also performed separately on individual populations [variance components (ii) and (iii)] to determine whether certain populations contributed more to the overall temporal component of variance. Results are reported for microsatellites only because this dataset analyzed DNA in all sampled trout and because similar analyses with SNPs reported congruent results (data not shown).

### Spatiotemporal analyses of putative adaptive genetic differentiation

We aimed to determine whether the 167 polymorphic SNPs located within transcribed regions of different coding genes, and exhibiting signatures of natural selection, were stable over time among Mistassini populations. To do so, we applied the Bayesian likelihood method implemented in BAYESCAN (Foll and Gaggiotti 2008) as it has two key advantages over other approaches: (i) It consistently shows the lowest false positive rates for detecting such outlier loci (Narum and Hess 2011; Vilas et al. 2012) and (ii) it does not assume an island model of gene flow to permit estimation of population-specific *F*_ST_. This latter characteristic was appropriate for Mistassini trout populations which exhibit gene flow asymmetries (Fraser et al. 2004). BAYESCAN estimates the probability that a locus is under selection by calculating the Bayes factor, the ratio of the posterior probabilities of two models (selection vs. neutral) given the data. Respectively, Bayes factors between 3 and 10 (log10 = 0.5–1), 10 and 32 (log10 = 1–1.5), 32 and 100 (log10 = 1.5–2), or exceeding 100 (log10 > 2), provide “substantial evidence”, “strong evidence”, “very strong evidence”, or “decisive evidence” of different statistical support for the two models, with posterior probabilities between 0.76 and 0.91, 0.91 and 0.97, 0.97 and 0.99, and >0.99.

For samples from each time period, we implemented 10 pilot runs of 10,000 iterations, a burn-in of 100,000 iterations, and 100,000 sampling iterations (sample size of 5000 and thinning interval of 20) to identify loci under selection. To evaluate the potential for loci to be under weaker selection, each genome scan was performed twice with different prior odds in assuming that a neutral model was, respectively, 5× and 10× more likely than a model of selection (the latter being more commonly used; Foll and Gaggiotti 2008). Given the similarly low number of SNPs detected as candidate outliers with both prior odds (5×, 2 SNPs; 10×, 1 SNP), results and inferences from these are based on genome scans using the 5× odds. For comparison, we further ran BAYESCAN across time periods for each population separately.

### Effective population size (*N*_*e*_) estimates

We employed the temporal and linkage disequilibrium methods to estimating contemporary generational *N*_*e*_ in each Mistassini population based on the microsatellite and SNP data separately. As both approaches assumed that selection does not cause allelic frequency change or linkage disequilibrium, in all calculations involving SNPs, two of 167 polymorphic SNP loci were removed because they were possibly under selection (see below).

First, we used a pseudo-likelihood temporal method assuming no gene flow (Wang 2001) to estimate *N*_*e*_, based on short-term allelic frequency changes between archival and contemporary time periods (archival data = 2000–2002 pooled for microsatellites). Although gene flow occurs among Mistassini populations, previous works on salmonids have found that the only available approach for estimating *N*_*e*_ that accounts for gene flow (Wang and Whitlock 2003) tends to underestimate *N*_*e*_ (Fraser et al. 2007a). Mistassini population *N*_*e*_ estimates were in fact 19–35% smaller when assuming gene flow than when it was not considered_,_ and whether or not gene flow was accounted for did not change the overall interpretation of *N*_*e*_ trends across populations (data not shown). Thus, only *N*_*e*_ estimates without gene flow are reported. Wang's (2001) method only allow whole integers for sampling intervals, so we carried out analyses with the following numbers of generations per sample (*T*′) (CHE = 2; PEP = 2; RUP = 3). We then converted the generated *N*_*e*_′ estimates to actual *N*_*e*_ using actual generation times of Mistassini populations (calculated from age data)*,* based on *N*_*e*_ = (*T*/*T*′) × *N*_*e*_′.

Second, *N*_*e*_ was estimated based on the single-sample linkage disequilibrium (LD*N*_*e*_) method of Waples and Do (2010), applied to each year's data for each population (microsatellites: total population samples = 12; SNPs: total population samples = 6). An advantage of this approach over the temporal methods above was that it could be used to estimate *N*_*e*_ in each time period and hence used to detect changes in population size over time. However, our samples were comprised of multiple cohorts. A random, mixed-age sample that includes a number of consecutive age classes of approximately one generation length should approximate a generational *N*_*e*_ estimate, but this has not been formally evaluated (Waples and Do 2010). We therefore assumed that any effect on *N*_*e*_ estimation caused by having mixed-age samples was equivalent across population samples. Note that low sample sizes per individual cohort – separated following aging of individual trout – also precluded *N*_*e*_ estimation based on individual cohorts.

### Traditional ecological knowledge

In September 2011 and August 2012, we collated local Cree knowledge on study populations, regarding changes observed between the years 2000 and 2011, based on (i) consultation meetings and dialogue with groups of fishers from the local community (where the number of individuals per meeting ranged from two to nine) and (ii) semi-directive interviews (*sensu* Nakashima 1990; Huntington 2000) with individual fishers. Interviewed fishers were guided in a discussion by the interviewer based on three general questions relating to trout population changes (Table 5). Fishers interviewed were the only local experts on trout in these localities for the time period of our research, according to the local Cree Trappers Association; typically, these were individuals with extensive guiding experience on the lake or rivers. A total of 14 individuals were interviewed: five for CHE/PEP and nine for RUP. Details of the advantages and disadvantages of our traditional knowledge collation techniques are discussed in Fraser et al. (2006). These techniques are sometimes viewed to be quantitatively difficult to interpret through the lens of western science with respect to sample size. Nevertheless, they are widely applied in remote areas, and provided that local experts are carefully chosen and screened (as in this study), they can play an important role in conservation and management decision-making, even if the information is derived from only several individuals (Huntington 2000; Fraser et al. 2006).

## Results

### Catch-per-unit effort, length, age, and length-at-age of prespawning trout

**Figure 2:**
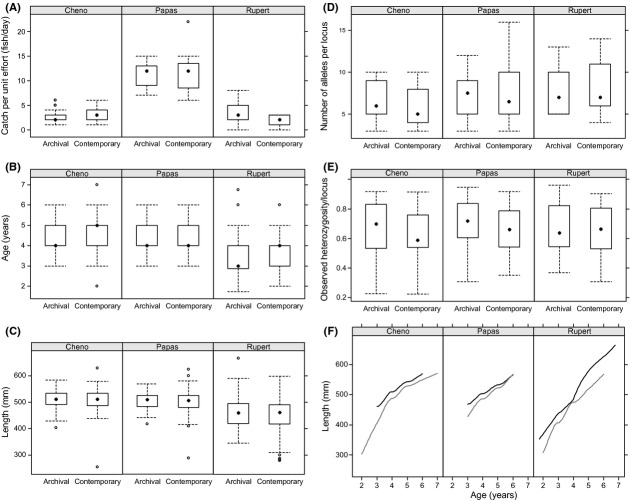
Trends in several metrics of population health between archival (2000–2002) and contemporary (2011) time periods within Mistassini Lake brook trout populations. Included are box plots for catch-per-unit effort (CPUE) (Panel A), length of prespawning trout (Panel B), age of prespawning trout (Panel C), the number of alleles per locus and observed heterozygosity at 14 microsatellite loci (Panels D and E), as well as smoothed (loess) plots of length-at-age for each population (black line for archival samples, gray line for contemporary) (Panel F). The lower and upper ends of each box represent the 25th and 75th quartiles, respectively. Medians are represented by the bold dots in each box. Skewness is reflected by the position of the median relative to the ends of each box. Whiskers extend from the top and bottom of each box to data no more than 1.5 times the interquartile range; values beyond this range (outliers) are represented by open circles.

For the GLM based on CPUE data, the interactive model with both time period (archival vs. current) and population was the best model (Table 1; both from an AIC perspective and based on the significance of parameter effect-sizes). PEP had higher CPUE than CHE or RUP in both time periods, but only RUP showed a difference between archival and contemporary periods, with lower CPUE in 2011 (Fig. 2).

**Table 1:** Results of two-factor generalized linear models (GLMs) to compare CPUE, length, age, length-at-age, and numbers of alleles per locus (allelic richness) and observed heterozygosities at 14 microsatellite loci, across time periods between and within Mistassini Lake brook trout populations.

Data	Model	Significant effects retained [Factor levels that differ]	AIC
CPUE	Population + Time	Population [PEP↑]	559.1
	Population * Time	Population [PEP↑], Population*Time[RUP-contemporary↓]	**552.5**
	Population	Population [PEP↑]	557.6
	Time		828.1
Length	Population + Time	Population [RUP↓]	4824.6
	Population * Time	Population [RUP↓]	4827.9
	**Population**	**Population [RUP↓]**	**4823.8**
	Time		4906.4
Age	Population + Time	Population [RUP↓]	1548.6
	Population * Time	Population [RUP↓]	1552.5
	**Population**	**Population [RUP↓]**	**1548.9**
	Time		1557.0
Length-at-age	**Population + Time**	**Time [contemporary↓], Population [RUP↑]**	**3733.3**
	Population * Time	Time [contemporary↓], Population [RUP↑]	3735.9
	Population	Population [RUP↑]	3787.8
	Time	Time [contemporary↓]	3769.9
Allelic richness	Population + Time		418.3
	Population * Time		421.7
	**Population**	**Population [RUP↑ vs. CHE]**	**416.2**
	Time		419.5
Heterozygosity	Population + Time		−46.2
	Population * Time		**−42.3**
	Population		−47.6
	Time		−49.9

For each metric analyzed, best-fit models are reported in bold, based on having lower AIC values. No significant effects were retained in any model based for observed heterozygosity.

There was no support for any effect of time period on length or age of prespawning trout, neither globally nor for individual populations (Table 1). RUP differed in average length and age, with shorter and younger fish, on average, than the other two similar populations (Fig. 2), but these differences were supported in both time periods. Similar results were obtained when sexes were analyzed separately (data not shown). The age composition (%) of each population was as follows (archival vs. contemporary periods): PEP (age 3+ to 6+: 18, 55, 21, 6 vs. 6, 45, 41, 8); CHE (age 2+ to 7+: 0, 18, 48, 28, 6, 0 vs. 2, 5, 38, 38, 15, 2); and RUP (age 2+ to 7+: 12, 40, 37, 7, 2, 2 vs. 6, 31, 44, 18, 1, 0).

Length-at-age varied with both time period and population, but not interactively. That is, across all populations, there was a smaller length-at-age ratio in contemporary than archival samples, implying a slower growth rate in recent years, although the trend was more pronounced in RUP (Table 1; Fig. 2). Average length-at-age was also higher in RUP (i.e., indicating faster growth) relative to the other two similar populations (Fig. 2).

### Within-population genetic diversity

All twelve population samples based on microsatellites (i.e., four samples × three populations) and all six population samples based on SNPs (two samples × three populations) were in HWE following Bonferroni correction, as evidenced by the low number of individual locus tests displaying significant departures from HWE with either a heterozygote excess (microsatellites: 2 of a total of 168 tests; SNPs: 37 of 1002 tests) or deficiency (microsatellites: 4 of a total of 168 tests; SNPs: 2 of 1002 tests). Departures were generally spread across different loci and population samples (Tables A1 and A2). There was little evidence of linkage disequilibrium between microsatellite loci within samples following Bonferroni correction (4 of a total of 1092 tests; 91 tests per population sample).

### Temporal analyses of genetic diversity and differentiation

All populations displayed similar amounts of genetic diversity over time: There was no support for any effect of time period on allelic richness or observed heterozygosity, neither globally nor interactively among populations, and only RUP showed slightly higher allelic richness than CHE (Tables 1, A1 and A2; Fig. 2). Among-population genetic structure was also stable over time periods, based on consistently greater differences in *θ*_ST_ among than within populations (Table 2), and a sixteen times lower temporal than spatial component of molecular variance (Table 3). In general, CHE and RUP showed the greatest temporal fluctuations, and PEP the least, in both *θ*_ST_ (Table 2) and in the amount of genetic variance explained temporally within each population (Table 3).

**Table 2:** Summary of spatiotemporal population genetic structure of Mistassini Lake brook trout populations based on the range of *θ*_ST_ values between and within populations, either within or between archival and contemporary time periods (where higher values indicate greater differentiation).

Archival period	Between or within populations		

	CHE	PEP	RUP
CHE	0.005–0.028 (2/3)	0.014–0.034 (9/9)	0.060–0.100 (9/9)
PEP		0.0006–0.008 (1/3)	0.053–0.081 (9/9)
RUP			0.002–0.013 (2/3)

Contemporary period	Between populations		

	CHE	PEP	RUP
CHE		0.014 (1/1)	0.066 (1/1)
PEP			0.065 (1/1)

Archival vs. Contemporary	Between or within populations		

	CHE archival	PEP archival	RUP archival
CHE contemporary	0.012–0.023 (3/3)	0.015–0.020 (3/3)	0.081–0.094 (3/3)
PEP contemporary		0.007–0.008 (2/3)	0.074–0.083 (3/3)
RUP contemporary			0.016–0.021 (3/3)

Fractions in parentheses represent the proportion of comparisons with statistically significant *θ*_ST_ values (*P* < 0.05).

**Table 3:** Hierarchical partitioning of genetic variance (AMOVA) at microsatellite loci among all Mistassini Lake brook trout populations and individual populations between sampling years.

Variance component	Among all Mistassini Lake populations		

	df	% Total variance	*P*
Among populations	2	6.32	***
Among time periods within populations	3	0.41	**
Within populations	1608	93.37	***

Variance component	Within individual populations		

	df	% Total variance	*P*
CHE			
Among time periods within populations	1	0.69	**
Within populations	374	99.31	***
PEP			
Among time periods within populations	1	0.18	0.19
Within populations	714	99.82	***
RUP			
Among time periods within populations	1	0.79	**
Within populations	546	99.21	***

Significance is shown at the **P = 0.01 or ***P = 0.001 level.

### Spatiotemporal trends in putative adaptive genetic differentiation

**Figure 3:**
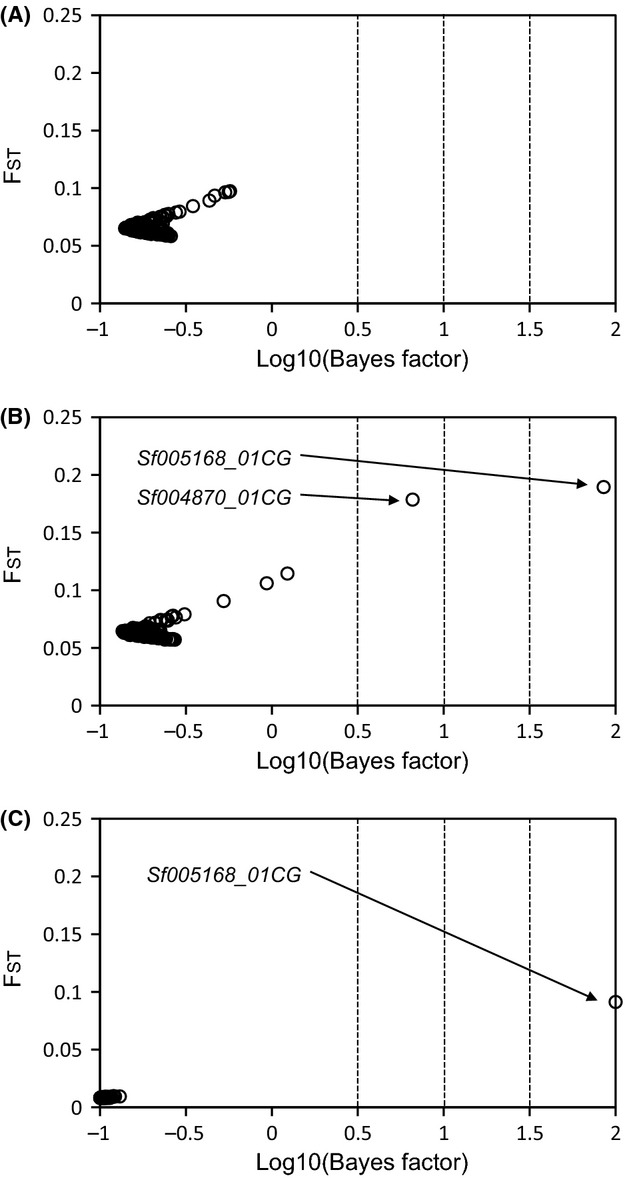
Spatial and temporal genome scans using BAYESCAN for identifying putative F_ST_ outlier SNP loci under selection. (A) Archival period (year 2000) among Mistassini populations. (B) Contemporary period (year 2011) among Mistassini populations. (C) Archival vs. contemporary period within the RUP population. Vertical dashed lines indicate log10(Bayes factor) values of 0.5, 1.0, and 1.5, corresponding to posterior probabilities of 0.76, 0.91, and 0.97, respectively. For standardizing axes, the log10(Bayes factor) value for the outlier SNP in panel “C” (RUP population) was denoted as 2 (its actual value was 1000).

Two SNPs screened were (i) found to be under directional selection in contemporary samples (Fig. 3); (ii) exhibited “substantial” (*Sf004870_01CG*) or “very strong” (*Sf005168_01CG*) probabilities (0.87, 0.99) of being under selection in BAYESCAN; and (iii) differentiated RUP from CHE and PEP. When genome scans were conducted on individual populations over time, changes in selection were also observed at *Sfo05168_01CG* in RUP (Fig. 3), with a “decisive” probability of 1.00 [log10(Bayes Factor) = 1000].

### Effective population size (*N*_*e*_) estimates

Estimates of *N*_*e*_ were consistently higher, and 95% CI were more often wider, with the single sample than the temporal method (Table 4). The 95% CI for *N*_*e*_ overlapped between microsatellites and SNPs with one exception (PEP), temporal method (Table 4). CHE likely has the smallest *N*_*e*_ of Mistassini populations and appears stable over time. PEP is a large *N*_*e*_ population with no apparent change over time. RUP is an intermediate to large *N*_*e*_ population, but its temporal stability is less clear. According to microsatellite data, this population might have experienced a contemporary decline in *N*_*e*_ given lower, nonoverlapping CI for *N*_*e*_ estimates in 2011 relative to two of three archival year *N*_*e*_ estimates (2000, 2002); according to SNP data, no temporal change in *N*_*e*_ has occurred.

**Table 4:** Estimates of *N*_*e*_ in Mistassini Lake brook trout populations based on one single-sample estimator (LD*N*_*e*_; Waples and Do 2010) and one “temporal” method (Wang 2001) and genetic markers.

Population sample	*μ*sat	SNP
**LD*****N***_***e***_		
CHE2000	241 (107–∞)	−675 (974–∞)
CHE2001	192 (106–705)	
CHE2002	61 (37–146)	
CHE2000–2002 harmonic mean	117	
CHE2011	236 (85–∞)	135 (99–205)
PEP2000	1241 (283–∞)	−500 (3912–∞)
PEP2001	−849 (360–∞)	
PEP2002	−213 (517–∞)	
PEP2000–2002 harmonic mean	449	
PEP2011	669 (357–3353)	3937 (549–∞)
RUP2000	−4087 (437–∞)	558 (237–∞)
RUP2001	−1433 (309–∞)	
RUP2002	−239 (929–∞)	
RUP2000–2002 harmonic mean	585	
RUP2011	200 (133–376)	426 (255–1198)
**Temporal** ***N***_***e***_		
CHE archival-contemporary	89 (59–160)	308 (120–∞)
PEP archival-contemporary	224 (154–363)	729 (404–∞)
RUP archival-contemporary	130 (96–186)	169 (93–500)

For LD*N*_*e*_ estimates, negative values are reported and not assumed to equal infinity; the 2000–2002 harmonic mean of *N*_*e*_ for microsatellite data incorporates these negative values (see Waples and Do 2010 for additional detail on interpreting negative values).

### Traditional ecological knowledge

Cree fishers noted some changes in the location and timing of trout captures in each river, as well as in the number of trout captured (Table 5). Later arrival of trout in the fall to each spawning river was noted for all populations. All RUP fishers noted changes in the spatial distribution of trout within this river, but no such distributional changes were noted in CHE/PEP. No changes in the capture success of CHE/PEP were noted by fishers, whereas more mixed responses were received by RUP fishers: Seven informants noted that no change in catch rate had occurred in the past decade, but two noted a slight decline (Table 5). A major concern expressed by Cree fishers regarding the future health of trout populations was the potential effect that climate change might have, particularly in relation to water temperature and water levels within RUP (Table 5).

**Table 5:** Summary of traditional ecological knowledge (TEK) of Mistassini Lake brook trout populations between 2000 and 2011.

General question	CHE	PEP	RUP
Where and when were trout found within rivers (where did fishing take place)?	Same locations as between 1970 and 2000* (1) Trout are returning to river later in the fall (1)	Same locations as between 1970 and 2000* (2) Trout are returning to river later in the fall (1)	Trout are changing locations and moving around more; fish are not being captured in the same places as before (9) Large trout are returning later to river in the fall (2)
Had the number of trout increased, decreased, or stayed the same over the past eleven years?	Same (2)	Same (3)	Same (7); Decreased slightly (2)
Did the informant have any concerns about the health of trout in the lake?	Intense fishing pressure of certain locations† (1)	Intense fishing pressure of certain locations† (1)	Climate change (increased river temperature, more variation in water levels) (6) Increased boating activity may be scaring the trout (3)

In parentheses is the number of interviewed Cree fishers making each general statement.

* Based on interviews with Cree Fishers from Fraser et al. (2006).

† Concern refers to the spatial location of harvesting within Mistassini Lake, not within rivers.

### General trends across population monitoring metrics

With the exception of reduced length-at-age, all metrics assessed were temporally stable in CHE and PEP (Table 6). In contrast, several metrics showed declines or shifts in contemporary RUP. Specifically, we found declines in length-at-age, CPUE, and *N*_*e*_ (relative to two of three archival years sampled, based on microsatellites; no decline based on SNPs), and evidence for a change in selection (based on one SNP not found in the archival period). There was also partial evidence for shifts in the RUP spatial distribution – and in a few cases, a catch reduction – noted by aboriginal fishers.

**Table 6:** Summary of the general, temporal trends between 2000 and 2011 across different monitoring metrics employed for each Mistassini brook trout population.

Metric	Detail	CHE	PEP	RUP
CPUE	According to western science	Stable	Stable	Declining
	According to traditional knowledge	Stable	Stable	Stable or declining
Habitat use	According to traditional knowledge	Stable	Stable	Shifting
Life history	Age	Stable	Stable	Stable
	Length	Stable	Stable	Stable
	Length-at-age	Declining	Declining	Declining
Genetic/genomic diversity	Heterozygosity	Stable	Stable	Stable
	Allelic richness	Stable	Stable	Stable
	Outlier loci	Stable	Stable	Shifting
*N*_*e*_		Stable	Stable	Stable or declining

Where applicable, confidence intervals for individual metrics had to be nonoverlapping between time periods to distinguish whether populations were stable, increasing, declining, or shifting. Trends in metrics based on traditional knowledge were derived from the general consensus across interviewed Cree fishers.

## Discussion

Our combination of multidisciplinary metrics revealed largely temporally stable characteristics over the past eleven years (2–3 generations) in two study populations (CHE, PEP), but a possible population shift and/or decline in a third population (RUP). Below, we illustrate how our approach is practical for guiding future population monitoring efforts in our study system under the precautionary principle. We further discuss some advantages and drawbacks to adopting a multidisciplinary approach to monitoring for other researchers to consider. But first, we consider whether the evidence for temporal change in one population reflects a demographic decline and some other key aspects of our results.

### Do changes in the adopted population metrics signal a demographic decline?

Although the detected changes in RUP could signal a possible demographic decline, the observed reduced length-at-age (particularly at older ages) and shifts at one SNP locus might reflect environmental variation and not be associated with fisheries-induced life-history change. We also acknowledge that many aboriginal fishers did not report reductions in catch rates. Their traditional knowledge suggested instead that reduced CPUE in 2011 might correspond to changes in RUP trout feeding locations or perhaps a contemporary trend for some adults to return later to spawn, and fishers expressed more concern about water temperature effects and levels in this river than about fishing pressure. So a similar temporal window of sampling across years (within 1–1.5 weeks) might not have been biologically the same for the returning spawning trout. Temporal shifts in trout spatiotemporal distribution and/or size distribution are plausible in RUP, for as the lake's outlet, it is a large and dynamic river influenced by an enormous catchment area, with many side channels filled with rapids and interconnected lakes. Nevertheless, we feel that such a temporal shift in returning spawning trout (perhaps due to increasing temperature) should not have affected the 2011 CPUE estimate because the vast majority of prespawning fish would have already entered this river by the time our sampling was conducted (Fraser et al. 2006). Finally, the possible reduction in RUP *N*_*e*_ may or may not reflect a reduction in adult census population size (*N*): (i) There is substantial variation in the positive relationship between *N*_*e*_ and *N* (Palstra and Fraser 2012), and the (ii) two parameters can have considerable independence over even a few generations due to processes that cause well-documented temporal changes to *N*_*e*_/*N* ratios (Ardren and Kapuscinski 2003; Shrimpton and Heath 2003; Fraser et al. 2007b). Overall, despite a lack of clear evidence for a demographic decline, we feel that it would be prudent for management to consider that present changes in RUP may be an indication of demographic change.

### Comments on *N*_*e*_ estimates and *N*_*e*_ estimation methods

The two most productive populations in Mistassini Lake in terms of total harvest (RUP, PEP) also had the most uncertainty in their *N*_*e*_ estimates, despite relatively large datasets (i.e., numbers of samples and loci). We contend that this uncertainty reflects that their *N*_*e*_ are (or were) in fact large, as suggested by the LD*N*_*e*_ method, rather than that the temporal *N*_*e*_ estimates are accurate. Disparities between these *N*_*e*_ estimation methods are not unexpected as they do not correspond to exactly the same time periods (Fraser et al. 2007a). Disparities might reflect: (i) a lack of precision for large populations or when samples are comprised of multiple cohorts (LD*N*_*e*_: Waples and Do 2010); (ii) downward biases in *N*_*e*_ estimation in large populations (temporal method: Waples 1990); and/or (iii) greater sampling noise in large populations, of note for RUP given local fisher descriptions of temporally changing spatial distributions (both methods).

### Inferring population abundance from genetic data

The ambiguity of some *N*_*e*_ estimates raises the question of what the actual abundances of this study's populations are, a critical consideration for ongoing local management. Given that (i) CHE *N*_*e*_ estimates were largely congruent across methods; (ii) CHE shares a similar life history, migration characteristics, and spatial habitat with PEP (Fraser and Bernatchez 2005); and (iii) CPUE was temporally stable within CHE and PEP, crude estimates of *N* for these two populations can be formulated based on *N*_*e*_/*N* ratios reported in other salmonid populations with analogous life histories (mean 0.17, range 0.06–0.31, from Heath et al. 2002 and Charlier et al. 2011). “Crude” is used here is to reemphasize that the relationship between *N*_*e*_ and *N* is not a strong one, particularly as *N*_*e*_ increases, and that salmonid fishes show substantial variation in *N*_*e*_/*N* ratios (Palstra and Fraser 2012).

Using a mean *N*_*e*_ of 100–200 in CHE, a range of *N*_*e*_/*N* ratios from 0.06 to 0.31 would generate a likely *N* of 323–3333 for this population; CPUE in PEP was consistently four or more times higher than that in CHE, so this would generate a range of *N* from 1292 to 13,332 in PEP. These values are consistent with current knowledge and observations on the low productivity of brook trout populations in large, oligotrophic lakes (Power 1980; Ridgway 2008). Additional genetic monitoring at least 2–3 generations from now (about 10–13 years), and/or a thorough mark–and–recapture study on each study population, would help to clarify their abundance (see below). We did not attempt to approximate *N* in RUP because this population does not share a similar life history, habitat, or spatial migration with the other Mistassini populations, or with other salmonid populations for which data are available (i.e., RUP has a potentially different *N*_*e*_/*N*).

### Temporal trends in putatively adaptive genetic differentiation

A low percentage of this study's SNPs were under possible selection compared with other studies (1.2% vs. 0.4–24.5%, average 8%; Strasburg et al. 2012). This weak evidence for putatively adaptive population genetic differentiation was unanticipated. First, local adaptation is strongly implicated at the scale between RUP and CHE/PEP based on many phenotypic and life-history differences (Fraser et al. 2004, 2005; Fraser and Bernatchez 2005), and on what is known in analogous salmonid populations (Fraser et al. 2011). The only outlier SNPs differentiated RUP, reinforcing the previous suggestion that local management should treat RUP and PEP/CHE separately (Fraser et al. 2006). Second, selection should have been easier to detect as each SNP was located within transcribed regions of a different coding gene (Sauvage et al. 2012a,b). Lamaze et al. (2012) reported a greater proportion of these same SNPs as outliers, but their study examined a scale encompassing more populations and likely greater environmental heterogeneity than this study. As many monitoring metrics were largely temporally stable in Mistassini Lake, perhaps selection at most traits (or linked loci) is weak within populations and more difficult to detect. The two outlier SNPs were not linked to any of 67 growth- and stress-related quantitative trait loci (QTL) identified in a recent linkage map for brook trout (Sauvage et al. 2012a,b). The outlier *Sf005168_01CG* codes for a protein (*Sox6,* a transcription factor) that may be responsible for maintaining muscle development in fish and other vertebrates (An et al. 2011), perhaps suggesting that environmental change or stress in the RUP might be impacting this in some way.

### Implications of using a multidisciplinary approach for future monitoring efforts

Had this study used only a couple of monitoring metrics or not involved local fisher perspectives in the monitoring process, potential shifts in RUP might not have been detected. The combined evidence (possible reduced CPUE and *N*_*e*_, and potential changes in local selective regimes) may provide an early warning sign that local management should be concerned with demographic changes in the population. We cannot confirm whether these shifts are related to fishing or to the environment, although we suspect the latter more so. To optimize monitoring, we have several recommendations for future sampling of these populations, based on this study's results, the strengths and weaknesses of different metrics, and bearing in mind that monitoring resources are typically limited.

First and foremost, we recommend that these populations be monitored every few generations to quantify catch rates and body size (length, age, length-at-age) based on western science and local aboriginal fisher knowledge independently. Changes in these metrics track environmental change very quickly (Jorgensen et al. 2007; Hutchings and Fraser 2008). These metrics are also more cost-effective to collect in our study region than genetic or mark–and–recapture studies. As a complement to this, specific environmental variables that might be linked to demographic change in RUP (water level, temperature) could further be monitored efficiently and cheaply through arrangements with local aboriginal fishers. Finally, gathering of local traditional knowledge might be improved beyond Fraser et al. (2006) and this study, by having local fishers record (catch) effort, location, fish size, and fish scales or otoliths (for aging). Overall, this collective information, repeatedly and independently obtained, would help to better discriminate whether or not populations are in decline and whether sampling biases are important, by allowing the application of, for example, approaches such as virtual population analysis (VPA; e.g., Pope 2002).

Nevertheless, changes to catch rates and body size or age do not generate an estimate of abundance of each trout population, a crucial parameter for mitigating potential overharvest signaled by reductions in these metrics. Therefore, if such reductions were detected, and additional resources were available, we suggest that either an additional genetic study or a thorough mark–and–recapture study would be useful for obtaining more confident abundance data than in this study, with the following caveats.

A genetic study would be most relevant if estimation of *N* from *N*_*e,*_ were improved, particularly when both these parameters are large (Tallmon et al. 2012; but see Côté et al. 2013). Such clarification is especially needed for species with relatively long generation times and when the timescale of monitoring is less than several generations. For example, recent simulations suggest that the LD*N*_*e*_ method does not correctly identify whether *N* is increasing or decreasing unless samples are spaced at least five generations apart (Tallmon et al. 2010). Encouragingly, Mistassini brook trout population genetic structure is now well established. There is a sufficiently large database of temporal samples from which to generate more accurate and precise trends in *N*_*e*_ in the future using more loci. New sample tissues could be easily collected alongside the body size data recommended above.

An intensive mark–and–recapture study would conversely provide the most concrete data on trout abundance. However, it could only be used to confidently estimate abundance for the most productive population in the lake's fishery (PEP). This river is more amenable to an effective tagging study than the other large population which has a low CPUE and inhabits a much larger, more complex habitat (RUP) (i.e., a labyrinth of side channels, stillwater and rapids), and the third population (CHE) which is accessible by bush plane and hiking only and also has a low CPUE.

Knowledge of the total annual harvest of brook trout (lake wide) would complement abundance data generated from either genetic or standard tagging data, for this information is currently unavailable. With several access points for nonlocal fishers and a growing local community of aboriginal fishers, such information might be best obtained from voluntary fisher surveys of effort and catch, with corrections accounting for the proportion of fishers that complete surveys.

### Advantages and disadvantages of multidisciplinary monitoring and general considerations

Our research was conducted in a truly remote setting where access was limited, the closest human settlement was 150–200 km away, and the only way to feasibly and humanely sample fish populations was via angling. In such circumstances, a pluralistic approach combining phenotypic and genetic approaches is a prudent one, given the inherent biological uncertainty involved in managing any harvested populations when demographic data are sparse and sample sizes are moderate at best. If multiple lines of evidence point to the same signal, they will provide more confidence in the result.

However, pluralistic studies need to be carefully interpreted, especially if there is some overlap in the samples used for each individual line of evidence. If multiple interpretations of results derive from the same biased sample, then one becomes more confident in a biased result. One might argue, for example, that some of our metrics were not truly independent, as the same individual samples were used to estimate changes in CPUE, life-history and genetic characteristics. Nonetheless, we feel that our sampling was largely free of bias, because each year within each population, samples were collected from many locations, were spread out temporally across and within days, and were obtained using the same angling techniques. In short, sampling designs in remote locations must be especially careful to avoid and account for biases wherever possible, by sampling at different times and locations and/or by comparing local resource user-based and researcher-based data.

Practically speaking, data for several of our monitoring metrics could be collected simultaneously in a relatively short time period. But we also acknowledge that pluralistic studies may demand more resources in some circumstances. An inherent trade-off may therefore exist between increasing the number of metrics adopted and ensuring reliable sample sizes. Is it better to collect only one or two data types to strictly adhere to sample size requirements? Or is it better to collect several types of data at perhaps suboptimal sample sizes, to represent multiple perspectives on the biological system? The former may improve precision but may not lead to more accuracy, whereas the latter may lead to more accuracy at the potential expense of being less precise. The best approach to take may depend on what information is critical to derive from population monitoring. Limited by sporadic sampling over a large geographic scale in Mistassini Lake, for example, a pluralistic approach pointed to a potential warning sign of demographic change in one population without using excessive resources. Now though, as alluded to above, it is critical to obtain more concrete information on population abundance: a future replication of the metrics employed in this study will only go so far with generating this required information, so management must consider whether additional resources are worth investing to generate more certainty.

A final caution for other researchers considering adopting a pluralistic approach to population monitoring is that even if data are rigorously collected, inconsistent results of multiple data types remain a real possibility. Yet we do not see this possible outcome as a disadvantage necessarily, as such inconsistency among data types may reflect true uncertainty in the biological system being studied. This only places a higher emphasis on the need for a precautionary approach to management decision-making.

## Data Accessibility

Data for this study are available at: datadryad.org.

## Appendix

Summaries of within-population genetic diversity at microsatellite and single nucleotide polymorphism (SNP) loci for each study brook trout population in Mistassini Lake, Quebec, Canada.

**Table A1:** Summary of within-population genetic diversity across fourteen microsatellite loci for each study brook trout population in Mistassini Lake, Quebec, Canada.

Population, year	*n*	Na (SE)	Ho (SE)	He (SE)	Loci with heterozygote deficiencies	Loci with heterozygote excesses
Cheno, 2000	49	5.70 (0.70)	0.67 (0.07)	0.65 (0.07)		
Cheno, 2001	58	6.46 (0.69)	0.67 (0.05)	0.64 (0.06)		
Cheno, 2002	30	5.46 (0.71)	0.61 (0.09)	0.62 (0.06)		
Cheno, 2011	41	5.79 (0.64)	0.62 (0.05)	0.65 (0.05)		
Pepeshquasati, 2000	72	7.87 (0.85)	0.71 (0.08)	0.69 (0.07)		*Sco220*
Pepeshquasati, 2001	69	7.68 (0.90)	0.70 (0.04)	0.74 (0.07)		
Pepeshquasati, 2002	44	7.22 (0.79)	0.63 (0.05)	0.62 (0.07)	*SfoB52*	
Pepeshquasati, 2011	173	7.42 (0.91)	0.65 (0.04)	0.66 (0.05)	*SfoB52, Ssa408*	
Rupert, 2000	78	8.01 (0.86)	0.64 (0.07)	0.68 (0.10)		*Sco218*
Rupert, 2001	50	7.82 (0.84)	0.63 (0.06)	0.62 (0.07)		
Rupert, 2002	50	7.95 (0.93)	0.60 (0.10)	0.59 (0.09)	*SfoD100*	
Rupert, 2011	96	7.93 (0.84)	0.65 (0.05)	0.64 (0.05)		

*n* = sample size, Na = mean number of alleles per locus; Ho = mean observed heterozygosity across loci; He = mean expected heterozygosity across loci. SE = 1 standard error.

**Table A2:** Summary of within-population genetic diversity across 167 polymorphic SNPs for each study brook trout population in Mistassini Lake, Quebec, Canada.

Population, year	*n*	Na (SE)	Ho (SE)	He (SE)	Loci with heterozygote deficiencies	Loci with heterozygote excesses
Cheno, 2000	37	1.90 (0.02)	0.32 (0.02)	0.29 (0.01)		*Sf000559_AG, Sf002439_CT, Sf004252_02GT, Sf004357_CT, Sf004416_AC, Sf004541_AC, Sf004560_GT*
Cheno, 2011	37	1.92 (0.02)	0.33 (0.02)	0.30 (0.01)	*Sf004938_CT*	*Sf000559_AG, Sf002439_CT, Sf004252_02CT, Sf004541_AC, Sf004541_AC, Sf004560GT*
Pepeshquasati, 2000	41	1.94 (0.02)	0.32 (0.02)	0.29 (0.01)		*Sf000905_02CT, Sf002439_CT, Sf004252_02GT, Sf004541_AC, Sf004560_GT*
Pepeshquasati, 2011	57	1.92 (0.02)	0.34 (0.02)	0.31 (0.01)		*Sf000559_AG, Sf004252_02GT, Sf004357_CT, Sf004541_AC, Sf004560_GT, Sf004416_AC*
Rupert, 2000	41	1.95 (0.02)	0.35 (0.02)	0.32 (0.01)		*Sf000854_01CT, Sf004357_CT, Sf004541_AC, Sf004765_01CG, Sf004975_01CG, Sf005186_CT*
Rupert, 2011	56	1.95 (0.02)	0.34 (0.02)	0.30 (0.01)	*Sf004155_001AG*	*Sf000505_CT, Sf002439_CT, Sf004252_02GT, Sf004357_CT, Sf004541_AC, Sf004560_GT, Sf004765_01CG*

*n* = sample size, Na = mean number of alleles per locus; Ho = mean observed heterozygosity across loci; He = mean expected heterozygosity across loci. SE = 1 standard error.
